# Pomegranate vinegar attenuates adiposity in obese rats through coordinated control of AMPK signaling in the liver and adipose tissue

**DOI:** 10.1186/1476-511X-12-163

**Published:** 2013-11-02

**Authors:** Elly Ok, Gyeong-Min Do, Yeni Lim, Ji-Eun Park, Yeo-Jin Park, Oran Kwon

**Affiliations:** 1 Department of Nutritional Science and Food Management, Ewha Womans University, 52, Ewhayeodae-gil, Seodaemun-gu, Seoul 120-750, Republic of Korea

**Keywords:** High-fat diet-induced obesity, Pomegranate vinegar, AMP-activated protein kinase, Hormone sensitive lipase, Fatty acid oxidation

## Abstract

**Background:**

The effect of pomegranate vinegar (PV) on adiposity was investigated in high-fat diet (HF)-induced obese rats.

**Methods:**

The rats were divided into 5 groups and treated with HF with PV or acetic acid (0, 6.5 or 13% w/w) for 16 weeks. Statistical analyses were performed by the Statistical Analysis Systems package, version 9.2.

**Results:**

Compared to control, PV supplementation increased phosphorylation of AMP-activated protein kinase (AMPK), leading to changes in mRNA expressions: increases for hormone sensitive lipase and mitochondrial uncoupling protein 2 and decreases for sterol regulatory element binding protein-1c (SREBP-1c) and peroxisome proliferator-activated receptorγ (PPARγ) in adipose tissue; increases for PPARα and carnitinepalmitoyltransferase-1a (CPT-1a) and decrease for SREBP-1c in the liver. Concomitantly, PV reduced increases of body weight (*p* = 0.048), fat mass (*p* = 0.033), hepatic triglycerides (*p* = 0.005), and plasma triglycerides (*p* = 0.001).

**Conclusions:**

These results suggest that PV attenuates adiposity through the coordinated control of AMPK, which leads to promotion of lipolysis in adipose tissue and stimulation of fatty acid oxidation in the liver.

## Background

Obesity is a chronic metabolic disorder that is characterized by excessive body fat and dysregulation of lipid metabolism. Hyperlipidemia in obesity is strongly associated with chronic diseases such as type 2 diabetes, cardiovascular disease, certain forms of cancer, and respiratory complications [1]. Currently two categories of medications are available to treat obesity: appetite suppressants and inhibitors of specific nutrient absorption. However, they often have undesirable side-effects. Therefore much attention has been focused on natural products, which may increase fat oxidation, decrease adipogenesis, and regulate lipid metabolism. AMP-activated protein kinase (AMPK) is regarded as important as it senses the cellular energy status and plays a critical role in the energy balance of the body through a concomitant inhibition of fatty acid synthesis and an activation of fatty acid oxidation [2]. Metabolic changes induced by AMPK involve both acute effects on the phosphorylation of key enzymes and chronic effects on the expression of genes involved in metabolic regulation [3]. Thus, AMPK has been recognized as a promising target for the management of obesity and hyperlipidemia.

Dietary acetic acid is metabolized to acetyl-CoA with the production of AMP [4], which, *in vitro*, results in the elevation of the AMP/ATP ratio and subsequent phosphorylation of AMPK [5]. Based on these findings, previous studies found that dietary acetic acid suppressed body fat accumulation in animals by regulating genes for energy consumption and fatty acid oxidation enzymes in liver [6]. However, the effects of dietary acetic acid on AMPK activation in adipose tissue remains relatively unexplored, although AMPK is ubiquitously expressed and plays an important role in various physiological and pathological processes in the liver and adipose tissue [7]. Furthermore, little information is available on the coordinated control of lipid metabolism through the phosphorylation of each AMPK protein and its downstream effectors in the liver and adipose tissue.

Finally, as many different kinds of vinegars are being introduced into the market, it is necessary to compare the relative bioactivities of newer vinegars. Pomegranate (*Punicagranatum L*.), a fruit native to the Middle East, and its juice and extracts are being widely promoted to consumers as a nutraceutical source. Therefore, this study was performed to compare the effects of new vinegar containing pomegranate extract (PV) with those of acetic acid on adiposity in high-fat diet-induced obese (DIO) rats. To provide mechanistic explanation, we also investigated the role of AMPK protein and its downstream effectors with a focus on the coordinated control of lipid metabolism between the liver and adipose tissue.

## Results and discussion

In this study, the effects of PV on adiposity were compared with those of acetic acids in DIO rats, which have characteristics of excess body fat, dyslipidemia, and fatty liver.

### Comparison of PV and acetic acid for attenuating adiposity in DIO rats

Changes in body weight, calorie intake, fat weight, and lipid profile in plasma and liver after a 16-week supplementation with PV or acetic acid are shown in Table 1. Although daily calorie intake was not different among the groups, both AH and VL supplementation significantly suppressed body weight increases induced by a high-fat diet (*p* = 0.048). Also AH or VL groups were also shown to decrease WAT. However, the dose-dependency was not clear in PV, resulting that changes in body weight and WAT were notably decreased in the VL group. Plasma triglyceride (TG) level was significantly lowered by acetic acid or PV supplementation compared with the HF control (*p* = 0.001), whereas plasma leptin level was tended to be decreased in the VL group only. Hepatic TG level was significantly lowered in the VL group versus the HF control (*p* = 0.005). These findings are consistent with data on body weight gain and adiposity.

**Table 1:** **Effects of acetic acid or pomegranate vinegar supplementation on the body weight gain, calorie intake**, **fat weight, and lipid profile in plasma and liver in Sprague Dawley rats fed a high fat diet for 16 weeks**

	**HF**	**AL**	**AH**	**VL**	**VH**
Body weight gain (g/16 weeks)	268.6 ± 10.6^a^	247.2 ± 10.3^ab^	230.5 ± 5.1^b^	235.3 ± 12.8^b^	259.6 ± 12.3^ab^
Calorie intake (kcal/day)	107.8 ± 2.2	104.1 ± 3.1	107.4 ± 1.5	104.1 ± 3.1	106.2 ± 1.8
WAT mass (g/100 g BW)					
Epididymal WAT	3.4 ± 0.1^a^	3.4 ± 0.0^ab^	3.3 ± 0.0^ab^	3.0 ± 0.1^b^	3.2 ± 0.1^ab^
Perirenal WAT	4.8 ± 0.2^a^	4.5 ± 0.1^ab^	4.3 ± 0.1^b^	4.3 ± 0.1^b^	4.4 ± 0.1^ab^
Plasma TG (mg/dL)	186.6 ± 8.9^a^	155.5 ± 10.8^b^	152.7 ± 8.1^b^	154.6 ± 10.4^b^	164.6 ± 10.1^b^
Plasma leptin (ng/mL)	10.2 ± 0.5	10.20 ± 0.9	9.9 ± 0.4	8.5 ± 0.4	9.84 ± 0.6
Hepatic TG (mg/g liver)	36.7 ± 4.5^a^	30.8 ± 3.0^ab^	30.0 ± 4.7^ab^	23.8 ± 1.9^b^	28.4 ± 3.1^ab^

Mean ± SEM (*n* = 10 per group). ^ab^Means not sharing common letters are significantly different between groups at *p* < 0.05 by Duncan’s multiple range tests. BW, body weight; WAT, white adipose tissue; TG, triglycerides; HF, high fat diet; AL, high fat with HF, high fat diet; AL, HF with low-dose acetic acid; AH, HF with high-dose acetic acid; VL, HF with low-dose PV; VH, HF with high-dose PV.

It is worth to note that the effects of PV on fat utilization in the liver and decrease in body weight and plasma triglycerides were more potent at the low-dose than the high-dose, but the effects of low-dose PV showed slightly more or equal potency than high-dose acetic acid. It might be attributed to the chemical composition of PV. However, a limitation of this study is that it was not designed to aim at tracing the causative components in PV due to the highly diverse phytochemicals found in PV. Further complicating matters, phytochemicals are transformed in the body into various metabolites after ingestion [8]. Future studies are needed to identify the principal bioactive components in PV, such as testing the activity of each fraction in a suitable cell culture model or high-throughput assay system [9]. In the meantime, ellagic acid that was recognized as having a potential role in contributing to altered gene expression by PV treatment in human hepatocyte in a less-specific approach [10] can be used as a marker compound for the purpose of standardizing PV.

### Effects of PV on the activation of AMPK and its downstream effectors in adipose tissue

**Figure 1:**
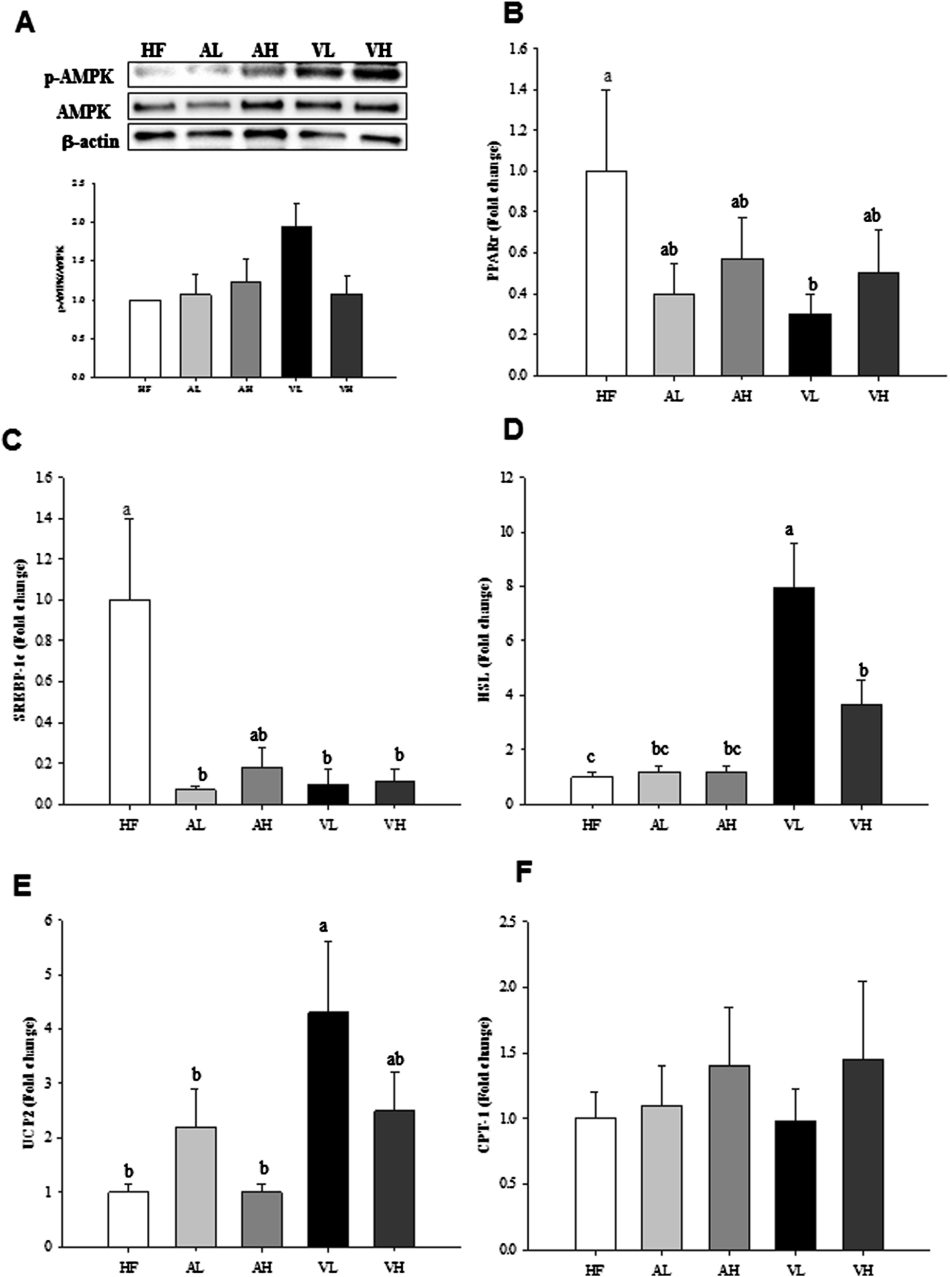
**Effects of pomegranate vinegar or acetic acid supplementation on the p-AMPK protein (A), PPARγ (B), SREBP-1c (C), HSL (D), UCP2 (E), CPT-1a (F) mRNA levels in adipose tissue of SD rats fed a HF diet for 16 weeks (*n*** **= 10 per group).** Representative western blots of adipose tissue lysate with antibodies against AMPK, p-AMPK and β-actin as loading control. Total protein (50 ug) from the adipose tissue was electrophoresed on to SDS-phage gel and transferred to PVDF membranes. The effects of dietary PV or acetic acid supplementation on key lipolysis enzyme mRNA levels were examined by real-time PCR. The results represent each mRNA/ β-actin expression ratio. Data are shown as the mean ± SEM. ^abc^Means not sharing common letters are significantly different between groups at *p* < 0.05.

Lipolysis in WAT is completed in a step-wise fashion initiated by adipose TG lipase and then hormone sensitive lipase (HSL) and monoacylglycerol lipase [11]. Existing literature indicates that high-fat diet feeding increased adipose TG lipase content in mouse, whereas activated HSL content was severely reduced [12], indicating that the hydrolysis of diacylglycerol by HSL is the rate-limiting step of WAT lipolysis [13]. It was also found that high-fat diet inhibited AMPK activation as well as PPARγ coactivator-1α expression, citrate synthase activity, and palmitate oxidation in WAT [12]. However, PV supplementation prevented high-fat diet induced changes in WAT as witnessed by up-regulation of HSL expression, down-regulation of SREBP-1c and PPARγ expression, and induction of AMPK activation in this study. Adipogenesis involves a highly regulated and coordinated cascade of transcription factors, such as members of the PPARs, the C/EBPs, and SREBP family, which together lead to the establishment of the differentiated state [14]. SREBP-1c, the most abundant form of SREBPs in adipose tissue and liver, controls the genes involved in lipogenesis. PPARγ also remarkably distributes in WAT and plays a crucial role in maintaining adipose expansion and adiposity [15]. AMPK signaling pathways have shown an inverse correlation with SREBP-1c as well as PPARγ in WAT [16]. Figure 1A shows the activaion of AMPK in WAT, indicating the phospho-AMPK levels in WAT were increased in the acetic acid and PV groups compared with the HF control. Expression of PPARγ mRNA was significantly decreased in VL group versus HF control (Figure 1B). In parallel with this, the expression of SREBP-1c mRNA was significantly decreased in the AL, VL, and VH groups compared to the HF control (Figure 1C). In contrast, the expression of HSL mRNA was significantly higher in the PV groups versus the HF control (Figure 1D). In particular, the VL group showed much greater increase in comparison to the HF control. The biological role of uncoupling protein2 (UCP2) is less clear, although some evidence suggests a role for this protein in energy balance and thermogenesis [17]. The results of the present study indicate that UCP2 mRNA was also significantly increased in the VL group only in comparison to the HF control (Figure 1E) in adipose tissue. However, CPT-1a, the rate-limiting enzyme of mitochondrial fatty acid oxidation, mRNA level was not changed with acetic acid or PV supplementation (Figure 1 F). Collectively, it appeared that PV promoted lipolysis as well as created milieu for preventing re-esterification of lipolytic products in WAT. Therefore, the net result was to favor lipid removal from WAT. The classical mechanism to explain these characteristics is highlighted by fact that dysregulation of lipolysis may lead to metabolic abnormalities [18,19].

### Effects of PV on the activation of AMPK and its downstream effectors in the liver

**Figure 2:**
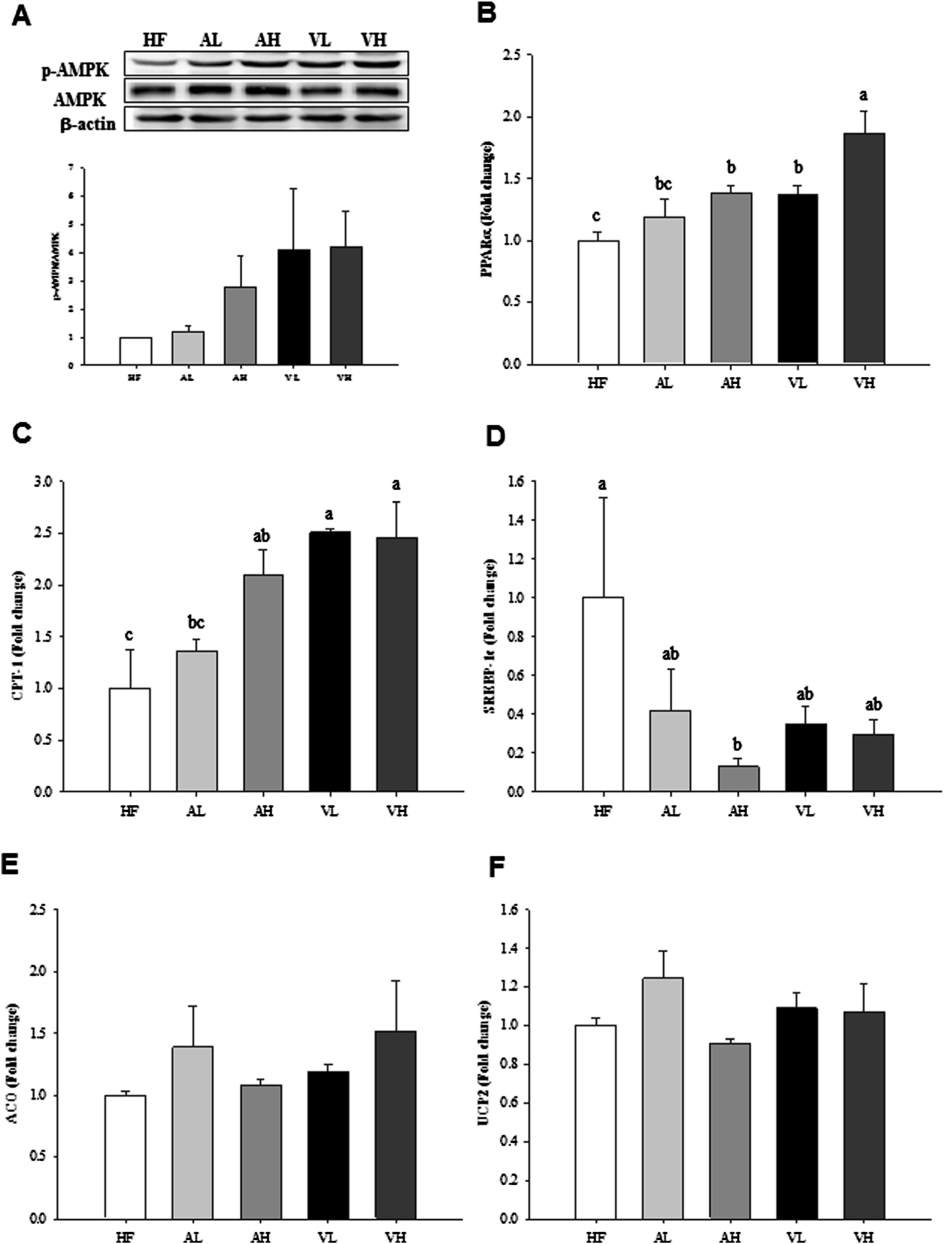
**Effects of pomegranate vinegar or acetic acid supplementation on the p-AMPK protein (A), PPARα (B), CPT-1a (C), SREBP-1c (D), ACO (E), UCP2 (F) mRNA levels in the liver of SD rats fed a HF diet for 16 weeks (*n*** **= 10 per group).** Representative western blots of adipose tissue lysate with antibodies against AMPK, p-AMPK and β-actin as loading control. Total protein (40 ug) from the liver was electrophoresed on to SDS-phage gel and transferred to PVDF membranes.The effects of dietary PV or acetic acid supplementation on hepatic key lipolysis enzyme mRNA levels were examined by real-time PCR. Data are shown as the mean ± SEM. ^abc^Means not sharing common letters are significantly different between groups at *p* < 0.05.

Abnormalities of peripheral lipid storage in obesity may result in overflow of lipid to the liver, leading to hepatic fat accumulation [20]. This study revealed that PV promoted fatty acid oxidation and inhibited lipogenesis in the liver as evidenced by the phosphorylation of AMPK as well as up-regulation of PPARα and CPT-1a expression and down-regulation of SREBP-1c expression. AMPK has been identified as a key regulator of lipid homeostasis in the liver [21], yielding a net effect of increasing fatty acid oxidation and diminishing glycerolipid synthesis. Figure 2A shows the increase in the AMPK phosphorylation after acetic acid or PV supplementation as compared to the HF control. In addition, the expression of both lipogenesis- and fatty acid oxidation-related genes was determined in the liver. The expressions of PPARα and CPT-1a mRNA were stimulated in the AH, VL, and VH group compared to the HF control (Figure 2B and C, respectively), while the expression of SREBP-1c mRNA, a transcription factor controlling lipogenesis, significantly declined only in the AH group, but tended to decrease in the AL, VL, and VH groups compared to the HF control (Figure 2D). The expressions of acyl-CoA oxidase (ACO) and UCP2 mRNA were not changed (Figure 2E and F, respectively). The effect of PV is not significantly different compared with that of acetic acid. These results are in agreement with the study by Kondo *et al.*[6] who found up-regulation of PPARα, ACO, CPT-1 and UCP2 genes after the addition of acetic acid to HepG2 cells. However, the up-regulation of PPARα and CPT-1a mRNA expressions and the phosphorylation of AMPK were more pronounced with PV than with acetic acid.

### Coordinated control of energy metabolism by PV in adipose tissue and liver

Vinegar intake reduced body weight, body fat mass, and serum triglyceride levels in obese Japanese subjects and attenuated lipid profile in diabetic rats [22,23]. In addition, dietary acetic acid reduces serum cholesterol and triacylglycerols in rats fed a cholesterol-rich diet [24]. In this study, PV showed higher potency than acetic acid in promoting lipolysis via increase of HSL mRNA in adipose tissue as well as fatty acid oxidation via increase of CPT-1a mRNA in liver. Taken together, our data support the potential health benefits of PV in coordinated control of energy metabolism through AMPK activation between liver and adipose tissue better than acetic acid. Especially, these findings, at molecular level, firmly proved that the low-dose PV exerted higher potency than the high-dose PV in adipose tissue. Adipose tissue stores excess energy under positive energy balance conditions, whereas it provides energy for other organs by releasing fatty acids under negative energy balance conditions. Excess fatty acids release from the adipose tissue may cause fatty acid overflux into the liver with the development of liver steatosis. The coordinated control of lipid metabolism between these two organs might be important to maximize the overall effectiveness of lipid disposal. It created an environment where the net metabolic effect is to favor lipid removal, which was associated with the interaction between p-AMPK-induced up-regulation of HSL mRNA and down-regulation of PPARγ and SREBP-1c mRNA in adipose tissue and AMPK-induced up-regulation of PPARα and CPT-1a mRNA and down-regulation of SREBP-1c mRNA in liver.

## Conclusions

Consequently, PV prevented hepatic lipid deposition by overflow of lipid to the liver resulting from abnormalities of peripheral lipid storage in HF status. It suggests that AMPK activation of PV might act as an energy sensor between liver and adipose tissue to improve metabolic health. Further study is warranted to investigate whether PV intake may help to protect dyslipidemia and to maintain a healthy body weight in overweight subjects.

## Methods

### Chemicals

Acetic acid and ellagic acid were purchased from Sigma-Aldrich (St. Louis, MO, USA). Antibodies (AMPK and phospho-AMPK) were purchased from Cell Signaling Technology (Denver, MA, USA). All solvents were purchased from Merck (Darmstadt, Germany).

### Materials

The PV was obtained from Daesang Corp. (Seoul, Korea). Briefly, pomegranate extract was added after the alcohol fermentation and then acetic acid fermentation was continued. The PV was standardized with acetic acid and ellagic acid by using high-performance liquid chromatography (HPLC, Agilent Technologies 1200, Santa Clara, CA, USA, Table 2). Acetic acid was analyzed using Aminex HPX-87H cation-exchange column (300 × 7.8 mm; Bio-Rad, Hercules, CA, USA) and a UV detector (210 nm); ellagic acid was analyzed using C18 Halo column (100 × 2.1 mm; HiChrom, Berkshire, UK) and a UV detector (360 nm) as described in our previous study [10].

**Table 2:** Composition of pomegranate vinegar

**(g/100 mL)**	**Pomegranate vinegar**
Oxalic acid	0.22
Citric acid	1.02
Malic acid	0.01
Succinic acid	0.06
Lactic acid	0.04
Total organic acid	0.84
Acetic acid	2.16
Total acid	4.6
Ellagic acid	0.001
Brix	29.3
pH	2.8

### Animal and diets

Ten-week-old male Sprague–Dawley rats were purchased from Jung-Ang Lab Animal Inc. (Seoul, Korea). The rats were housed individually with a 12 h light/dark cycle at a temperature of 23 ± 1°C and a humidity of 45 ± 5% with access to water and chow diet(Samyang Co, Incheon, Korea) for a week prior to the experiment. For the experiment, rats were randomly divided into five groups (*n* = 10 for each) and fed the designated experimental diet (Table 3) for 16 weeks: high-fat diet (HF; modified AIN-93G diet containing 41.2% energy from fat), low-dose acetic acid (AL; HF with 6.5% (w/w) acetic acid; equivalent to 1.6% acetic acid per rat), high-dose acetic acid (AH; HF with 13% (w/w) acetic acid; equivalent to 3.2% acetic acid per rat), low-dose PV (VL; HF with 6.5% (w/w) PV; equivalent to 1.62% PV per rat) and high-dose PV group (VH; HF with 13% (w/w) PV; equivalent to 3.2% acetic acid per rat).

**Table 3:** Composition of experimental diets

**(g/kg)**	**HF**	**AL**	**AH**	**VL**	**VH**
Cornstarch	290.6	290.6	290.6	290.6	290.6
Dextrinized cornstarch	90.0	90.0	90.0	90.0	90.0
Sucrose	70.0	70.0	70.0	70.0	70.0
Casein (>85% protein)	100.0	100.0	100.0	100.0	100.0
Soybean oil	100.0	100.0	100.0	100.0	100.0
Lard	230.0	230.0	230.0	230.0	230.0
Fiber	60.0	60.0	60.0	60.0	60.0
Mineral mix^a^	41.0	41.0	41.0	41.0	41.0
Vitamin mix^b^	12.0	12.0	12.0	12.0	12.0
L-Cystine	3.5	3.5	3.5	3.5	3.5
Choline bitartrate	2.9	2.9	2.9	2.9	2.9
Tert-butylhydroquinone	0.0	0.0	0.0	0.0	0.0
Distilled water	150.0	75.0	0.0	75.0	0.0
Pomegranate vinegar (PV)	0.0	0.0	0.0	75.0	150.0
Acetic acid	0.0	75.0	150.0	0.0	0.0
**Total amount**	1,150.0	1,150.0	1,150.0	1,150.0	1,150.0
**Total calories (kcal)**	4372.7	4372.7	4372.7	4372.7	4372.7
Carbohydrates (% as kcal)	39.7	39.7	39.7	39.7	39.7
Protein (% as kcal)	19.1	19.1	19.1	19.1	19.1
Fat (% as kcal)	41.2	41.2	41.2	41.2	41.2

HF, high fat diet; AL, HF with low-dose acetic acid; AH, HF with high-dose acetic acid; VL, HF with low-dose PV; VH, HF with high-dose PV.

^a^Mineral mix (AIN-93G-MIX, g/kg mixture).

^b^Vitamin mix (AIN-93G-MIX, g/kg mixture).

The AL and AH group contained the same amount of acetic acid as the VL and VH group, respectively. The doses were determined on the basis of the previously published studies [6,25]. Body weights and food intakes were recorded weekly. Calorie intakes toward daily intakes were also converted. After the 16-week study period, liver and white adipose tissue (WAT: epididymal and perirenal fat pads) were removed in an overnight fasting state and stored at -80°C before use. Blood was also collected and quickly centrifuged at 4°C for 10 min. The serum fraction was collected and stored at -80°C for later analysis. The experimental protocol was approved by the Institutional Animal Care and Use Committee (IACUC) at Ewha Womans University (Reference # 2010-14-1).

### Biochemical assays

Plasma and hepatic TG were measured enzymatically using commercially available assay kits (Asan Pharm, Hwasung, Korea). For determination of hepatic TG content, liver tissue was homogenized and then total lipid was extracted by Bligh’s method [26]. Plasma leptin was measured using a radioimmunoassay kit (Invitrogen, Carlsbad, CA, USA).

### Quantitative TaqMan reverse transcription polymerase chain reaction analysis

Total RNA was extracted from liver and adipose tissue using TRIZOL (Invitrogen Co., Carlsbad, CA, USA). RNA concentration and quality were determined by a BioSpec-nano (Shimadzu Corp., Kyoto, Japan). cDNA was constructed using the High Capacity RNA-to-cDNA kit (Applied Biosystems, Foster City, CA, USA). Quantitative RT-PCR was performed using the TaqMan method in a Step-One-Plus RT-PCR System (Applied Biosystems). The primer sets for target genes were PPARα (Rn00566193_m1), SREBP-1c (Rn01446560_m1), PPARγ (Rn00440940_m1), ACO (Rn00569216_m1), CPT-1a (Rn00580702_m1), HSL (Rn00563444_m1), UCP2 (Rn01754856_m1) and β-actin (Rn00667869_m1). The relative amounts of these mRNAs were normalized to the amount of β-actin and the relative amounts of all RNAs were calculated using the comparative C_T_ Method [27].

### Western blot analysis

Liver and adipose tissue protein was extracted with lysis buffer (Intron Biotech, Seoul, Korea) and quantified using the Bradford method. Equal amount of proteins were electrophoresed using 0.1% SDS-polyacrylamide gel, transferred to polyvinylidenedifluoride membranes (Bio-Rad, CA, USA), incubated with 5% skimmed milk in Tris-buffered saline, and treated with rabbit anti-p-AMPK or rabbit anti-AMPK and mouse anti-β-actin (Santa Cruz Biotechnology, Santa Cruz, CA, USA). The immunoreactive antigen was then recognized by using a horseradish peroxidase-labeled anti-rabbit or anti-mouse IgG (Santa Cruz Biotechnology). Immunoreactive protein bands were visualized by ChemiDoc XRS System (Bio-Rad, CA, USA).

### Statistical analysis

Results were presented as mean ± standard error of mean (SEM). Statistical analyses were performed by the Statistical Analysis Systems package, version 9.2 (SAS Institute, Cary, NY, USA). The differences between treated groups were analyzed by one-way analysis of variance (ANOVA) with post hoc Duncan’s multiple range tests. Results were considered statistically significant at *p* < 0.05.

## Abbreviations

ACO: Acyl-CoA oxidase; AMPK: AMP-activated protein kinase; CPT-1: Carnitinepalmitoyltransferase-1; HF: High-fat diet; HSL: Hormone sensitive lipase; PPAR: Peroxisome proliferator-activated receptor; PV: Pomegranate vinegar; SREBP-1c: Sterol regulatory element binding protein-1c; TG: Triglyceride; UCP-2: Uncoupling protein-2; WAT: White adipose tissue.

## Competing interests

None of the authors have any competing interests.

## Authors’ contributions

This paper has been read and approved by all authors. EO, YL, JEP, YJP contributed to data analysis and execution of the experimental procedure; GM do contributed to data interpretation and preparation of the manuscript; OK won was responsible for the conception and design of the study, obtaining funding, and performed substantial editing of the manuscript.
